# The evolutionary origin of the universal distribution of mutation fitness effect

**DOI:** 10.1371/journal.pcbi.1008822

**Published:** 2021-03-08

**Authors:** Ayuna Barlukova, Igor M. Rouzine

**Affiliations:** Sorbonne Université, Institute de Biologie Paris-Seine, Laboratoire de Biologie Computationnelle et Quantitative, Paris, France; University of Ottawa, CANADA

## Abstract

An intriguing fact long defying explanation is the observation of a universal exponential distribution of beneficial mutations in fitness effect for different microorganisms. To explain this effect, we use a population model including mutation, directional selection, linkage, and genetic drift. The multiple-mutation regime of adaptation at large population sizes (traveling wave regime) is considered. We demonstrate analytically and by simulation that, regardless of the inherent distribution of mutation fitness effect across genomic sites, an exponential distribution of fitness effects emerges in the long term. This result follows from the exponential statistics of the frequency of the less-fit alleles, *f*, that we predict to evolve, in the long term, for both polymorphic and monomorphic sites. We map the logarithmic slope of the distribution onto the previously derived fixation probability and demonstrate that it increases linearly in time. Our results demonstrate a striking difference between the distribution of fitness effects observed experimentally for naturally occurring mutations, and the "inherent" distribution obtained in a directed-mutagenesis experiment, which can have any shape depending on the organism. Based on these results, we develop a new method to measure the fitness effect of mutations for each variable residue using DNA sequences sampled from adapting populations. This new method is not sensitive to linkage effects and does not require the one-site model assumptions.

## Author summary

The spectrum of beneficial mutations in their fitness effect observed in an adaptation experiment has often an exponential form. This shape is not observed for deleterious mutations. To explain this remarkable empiric fact, we use a model of evolving population, which includes mutation, natural selection of many linked loci, and genetic drift. We demonstrate that, regardless of the inherent distribution of the fitness effect across genomic sites, an observable exponential distribution emerges in the long term, as a consequence of the evolutionary process. We show that the logarithmic slope of this distribution increases in time. Based on these results, we develop a new method to measure fitness effects of mutations for variable residues using DNA sequences sampled from adapting populations.

## Introduction

Evolutionary dynamics of a population of nucleic acid sequences is controlled by several acting forces, including random mutation, natural selection, genetic drift, and linkage opposed by recombination. Of central interest is the adaptation of an organism to a new environment, which occurs due to the fixation in a population of rare mutations that increase the fitness of the organism [1–5]. The existing models with directional selection and adaptation in a multi-site population demonstrate that only those beneficial mutations that are established in a population, as opposed to those becoming extinct, contribute to the average speed of adaptation in the long term. The advantage of each favorable mutation is measured by the relative change it causes in genome fitness (average progeny number). Thus, the knowledge of fitness effects for different mutations is essential for predicting the evolutionary trajectory of a population, for example, during the development of resistance of a pathogen to treatment or the immune response. Therefore, a great effort has been invested in their estimation.

In the HIV genome, the average-over-genome fitness effect of a beneficial mutation, ~1%, was estimated using genetic samples from infected patients [6]. Finding out the Distribution of Fitness Effects of new mutations (DFE) over genomic sites in viruses and bacteria requires specially designed and elaborate experiments [1–5]. Selection coefficients for different sites of the hemagglutinin gene of human influenza A/H3N2 were estimated by fitting the deterministic one-locus model and its approximate extension for two-loci [7], where the model was fit to time-series data on allele frequencies. Another group [8] proposed a method of DFE estimation for deleterious mutations in mutation-selection-drift equilibrium based on the assumption that DFE has the form of the gamma distribution. These efforts emphasize the need for a more general approach based on evolutionary dynamics and not restricted to a one-locus model [9].

A major complication in predicting an evolutionary trajectory and estimating mutational effects is that the fates of individual alleles at different genomic sites are not independent due to clonal interference and linkage effects [10,11]. These effects increase with the number of linked variable sites. Another factor creating site-site interference is epistasis [12,13]. Recent advances in theoretical population genetics provide accurate and general expressions for the average speed of adaptation of an asexual population, as well as well for other observable parameters, such as genetic diversity, the probability of allele fixation, and phylogenetic properties. The technique is the traveling wave theory [14–28]. These models show that the evolution of a multi-site genome can be described by a narrow distribution of genomes in fitness, which slowly moves towards higher or lower fitness. The speed and direction depend on the interplay between selection, mutation, random drift, and linkage effects and recombination. The traveling wave was observed experimentally in yeast [29]. In all these models, the distribution of fitness effects across mutation sites (DFE) serves as an important input parameter, in addition to the population size, mutation rate, and recombination rate.

**Fig 1:**
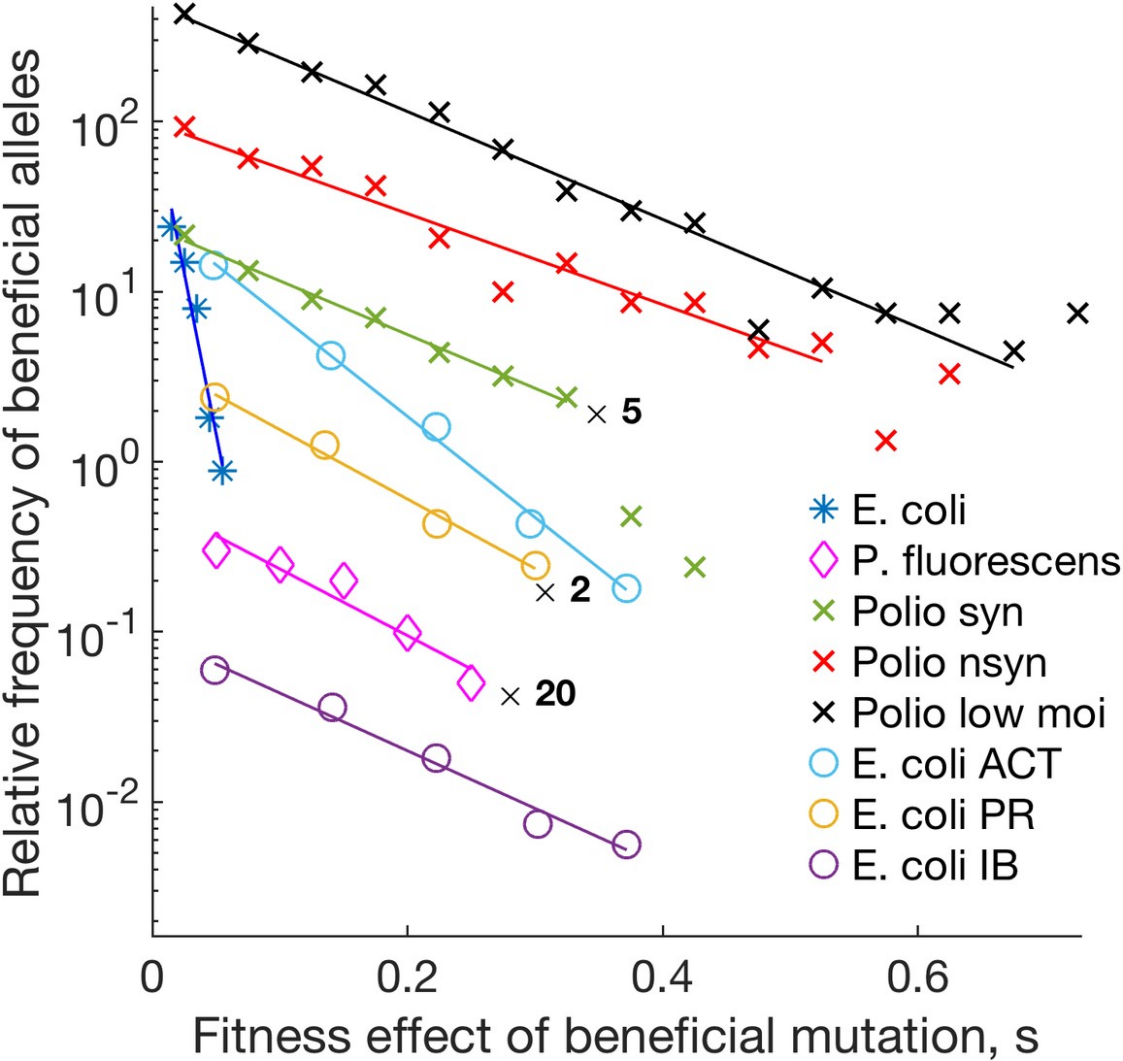
Different studies on distribution of fitness effects of beneficial mutations demonstrate an exponential form. Y-axis: Frequency of beneficial alleles (arbitrary units), *DFE*(*s*,*t*)*π*(*s*) in Eq 1. X-axis: Mutation gain in fitness due to a beneficial mutation (selection coefficient). Symbols represent results obtained for different sites of the genome in experiments on *Escherichia coli* [1], *Pseudomonas fluorescens* [2], poliovirus synonymous mutations, poliovirus non-synonymous mutations [3], poliovirus low MOI [4], *E*. *coli* acetamide (ACT), propionamide (PR), and isobutyramide (IB) [5].

In the present work, we propose a rather general approach to measure selection coefficients for specific sites that applies in the presence of multi-site linkage, both within and outside of the traveling wave regime. The key to the method is the intriguing fact that DFE for beneficial mutations has frequently an exponential form, which was observed for *E*. *coli*, *Pseudomonas aeruginosa*, *Pseudomonas fluorescence*, and poliovirus (Fig 1) [1–5]. We offer a simple interpretation of this phenomenon. We demonstrate that, regardless of the initial distribution of fitness effects across genomic sites, an exponential DFE emerges naturally, as a consequence of the evolutionary process of slow adaptation. However, the prediction is not completely universal. When the population approaches equilibrium, this result ceases to apply. Based on these findings, we develop a method of estimating the fitness effect of mutation for each variable site in the genome.

In the existing literature on DFE, two different distributions are referred as DFE. The first is the inherent distribution of selection coefficients of a genome, which represents the genome site density with respect to the values of their selection coefficient. This distribution can be observed in a site-directed mutagenesis experiment, where the fitness difference between alleles for each site is measured [30,31]. We will refer to it as "intrinsic DFE" to emphasize the fact that it is the property of the pathogen/environment and does not depend on the state of population. Another distribution is the distribution of new beneficial mutations arising naturally in an evolution experiment, which depends on the state of adapting population (Fig 1). We will use term "DFE" to denote the second distribution. We demonstrate below that these two distributions are different from each other [32], and that only one of them is close to the exponential. We focus on beneficial mutations only.

## Results

In order to explain the exponential shape of DFE observed in the experiments, we start by noting that beneficial mutations can emerge only at the sites currently occupied with less-fit alleles. Here and below we assume binary genomes with only two alleles per site: the best-fit and the less-fit. Although each position, in principle, can have four nucleotides A, C, T, G, in real viral data, on moderate time scales 1–10,000 generations, most variable sites display only two alleles in a sample. If a genomic site is occupied by the less-fit allele, it can become the best-fit by mutation, and a genomic site occupied by the best-fit allele can only lose in fitness by mutation. If a population is well-adapted during the process of evolution, most of genome sites, in each genome, already carry best-fit alleles and cannot experience beneficial mutations. Therefore, the observed DFE will be affected by the occupation number distribution of less-fit alleles among sites with different *s*, i.e., by the state of population, which depends on time, *t*.

Let denote the average frequency of less-fit alleles at a site with fitness effect *s* by *f*(*s*,*t*). We note that *f*(*s*,*t*) can also be viewed as the frequency of sites available for beneficial mutations. For example, consider a sequence of the form 1000001, where 1 stands for the less-fit allele and 0 for the best-fit allele. Then, only the first and the last positions in the sequence are the sites where a beneficial mutation can occur, 1→0. Thus, the rate of beneficial mutation at any fixed position of the genome must be proportional to the frequency of less-fit allele, *f*, at this position. If the system is fully adapted, we have *f* = 0, and no beneficial mutations are possible.

### Experiment description

Experiments shown in Fig 1 count beneficial mutations naturally emerging in an adapting population. The authors evolve a population of bacteria or virus for a short time in culture. Newly emerging beneficial mutations result in spontaneous increase in the best-fit allele frequency in time (selection sweeps). Although exact protocols differ, the count is done for naturally occurring mutations, not for random mutagenesis. In one experiment [3], the researchers used a deep sequencing technique CirSeq to monitor the arising frequency of minority alleles at each genomic site as a function of time and fit it with a simple one-site evolution model expression to estimate *s* for each site. Another group [1] focused on beneficial mutations in *E*. *Coli*. They measured selection coefficient *s* for each selection sweep from time series, and then counted the number of sweeps at sites belonging to an interval of the selection coefficient (*X*-axis in Fig 1). Therefore, all these experiments measure the naturally occurring mutation density, and not intrinsic DFE.

In experiments on natural evolution in Fig 1, beneficial mutation events with fitness gain *s* occur spontaneously at rare less-fit sites. If a new allele is lucky to be established in a population, it becomes fixed later in a deterministic fashion. Let function *φ*(*s*) denote the establishment probability of an allele with fitness benefit *s*. Experiments in Fig 1 detect established mutations with observable density *DFE*(*s*,*t*)*φ*(*s*), where

DFE(s,t)≡f(s,t)g(s) (1)

Here *f*(*s*,*t*) denotes the frequency of target sites available for beneficial mutations averaged over realizations (experimental replicas or independent populations from a statistical ensemble), and "intrinsic" quantity *g*(*s*)*ds* is the density of sites with the selection coefficient in interval [*s*, *s*+*ds*].

Functions *g*(*s*) and *f*(*s*,*t*) have different biological meaning [32]. One of them, *f*(*s*,*t*), depends on the state of population and time. Another, *g*(*s*), does not. Intrinsic distribution *g*(*s*) is a property of the virus and the cell culture and does not depend on time, nor it depends on whether a site is occupied by a better-fit or less-fit allele. It is expected to vary broadly between viruses and proteins. For example, some proteins may be more conserved and some less, and *g*(*s*) would be shifted towards larger and smaller s, respectively. Parameter *g*(*s*) is measured in experiment by performing site mutagenesis for each site, one by one, and evaluating fitness differences between the wild type and mutant strains (for example, by growth competition experiment) [30,31]. In contrast, the value in Fig 1

DFE(s,t)φ(s)=f(s,t)g(s)φ(s) (2)

is measured by actually evolving the virus in a culture and counting naturally arising mutations in an interval of *s*. The two distributions differ, because, in the evolution experiment, a beneficial mutation cannot arise if the site is already occupied with a best-fit allele. In other words, DFE depends on the ensemble-average probability of deleterious allele, *f*(*s*,*t*), and evolves in time.

Below mutant frequency *f*(*s*,*t*) is assumed to have pre-evolved before the experiment for a long time, reflecting the pre-history of the population under similar conditions. We also assume that the population is not in mutation-selection drift equilibrium yet, i.e., it is not best adapted to the conditions of the experiment. *f*(*s*,*t*) represents the ensemble average for a site. The initial genome in each experiment in Fig 1 is obtained, originally, by sampling from a previous, well-evolved population, close to the best-fit sequence. Then, an experimentalist stores the sample in the freezer. Later the virus is thawed and expanded in the culture, and then it evolves and *s* is measured for spontaneous mutations. Hence, the frequency of uniformly deleterious sites in the initial uniform population an experiment genome mimics the occupancy probability in the previous population. We will describe this pre-evolution of *f*(*s*,*t*) by simulations and analytically. After predicting the form of *f*(*s*,*t*) we will use it to estimate intrinsic distribution of *g*(*s*) from data.

We will show below that *f*(*s*,*t*) depends sharply (exponentially) on *s*, and the log slope of the dependence of *f*(*s*,*t*) on *s* increases linearly in time. The scale of *g*(*s*) in *s* stays constant. Therefore, sooner or later, exponential *f*(*s*,*t*) will change with *s* more sharply than *g*(*s*). It is a well-known mathematical fact that an exponential *f*(*s*,*t*) multiplied by a slower function *g*(*s*) still appears to be an exponential in the log plot. Therefore, the measured DFE appears as an exponential in the log scale, which explains the experimental results (Fig 1).

### Model

We consider an asexual organism, which evolved for some time but is still far from the mutation-selection equilibrium before the experiment. A haploid population has *N* binary sequences, where each genome site (nucleotide position) numbered by *i* = 1,2,…,*L* carries one of two possible genetic variants (alleles), denoted *K*_*i*_ = 0 or *K*_*i*_ = 1. Each site (nucleotide position) has one of two alleles: the better-fit (for example, A), or the less-fit (for example, G). We focus here on the moderate-term adaptation to a new constant environment, where the bi-allelic model is a fair approximation.

The genome is assumed to be very long, *L*≫1. Time is discrete and measured in population generations. The evolution of the population is described by a standard Wright-Fisher model, which includes the factors of random mutation with genomic rate *μL*, constant directional selection, and random genetic drift. Recombination is assumed to be absent. We do not consider balancing selection, diploid immune dominance, or selection for diversity. Once per generation, each individual genome is replaced by a random number of its progeny which obeys multinomial distribution. The total population size stays constant. To include directional natural selection, the average progeny number (fitness) of sequence {*K*_*i*_} is denoted *e*^*W*^, where the fitness effects of mutations, *s*_*i*_, are additive over sites

$$W=-\sum_{i=1}^{L}s_{i}K_{i}$$ (3)

The reference genome, {*K*_*i*_}≡0, can be chosen in an arbitrary way. For our aim, it is convenient to chose it as the best-fit sequence, so that all *s*_*i*_>0. Each site *i* with the deleterious allele, *K*_*i*_ = 1, is a target site for a possible beneficial mutation. Conversely, a site with the favorable allele, *K*_*i*_ = 0, can have a deleterious mutation.

The present model does not consider epistasis and assumes additive contributions of single sites to the fitness landscape. Our rationale for not considering epistasis explicitly is that, in the present work, we consider a short-term dynamics, in which most epistatic interactions are incorporated in the current values of *s*. If protein evolves for a very long time, a large part of its sequence will change, each mutation will trigger changes in other sites, and these changes become permanent and effect the subsequent values of *s* [6,33,34]. In the long-term, epistasis redefines the values of *s*. Therefore, on very long time scales, when many sites experience replacement of an allele, interaction of each site with many other sites has to be considered. On a short time scale, few sites are polymorphic and even fewer interact, so that not including epistasis is a fair approximation for most sites. Epistatic interactions with monomorphic sites are embedded in the values of *s*. A more general model of pairwise interactions is analyzed in [12,13,35] and, for global epistasis, in [36].

The fitness cost of a deleterious allele *s* is distributed in a complex way among genomic sites. In general, inherent distribution *g*(*s*) is unknown and depends on a virus, host cell type, and protein. Below we make no assumptions regarding the form of *g*(*s*) and demonstrate that the exponential shape in the less-fit allele frequency 〈*f*(*s*,*t*)〉 arises automatically and independently of the form of *g*(*s*). Later on, we will show how *g*(*s*) can be calculated from sequence data. We note again that our approach applies only far from mutation-selection equilibrium, when the system is still adapting. It is well known that, in equilibrium, the dependence 〈*f*(*s*,*t*)〉 on *s* is not exponential, but close to *f* = *μ*/*s* for infinite *N* [9] and a more complex dependence for finite *N* [15]. This is the effect of deleterious mutations.

### Monte-Carlo simulation

**Fig 2:**
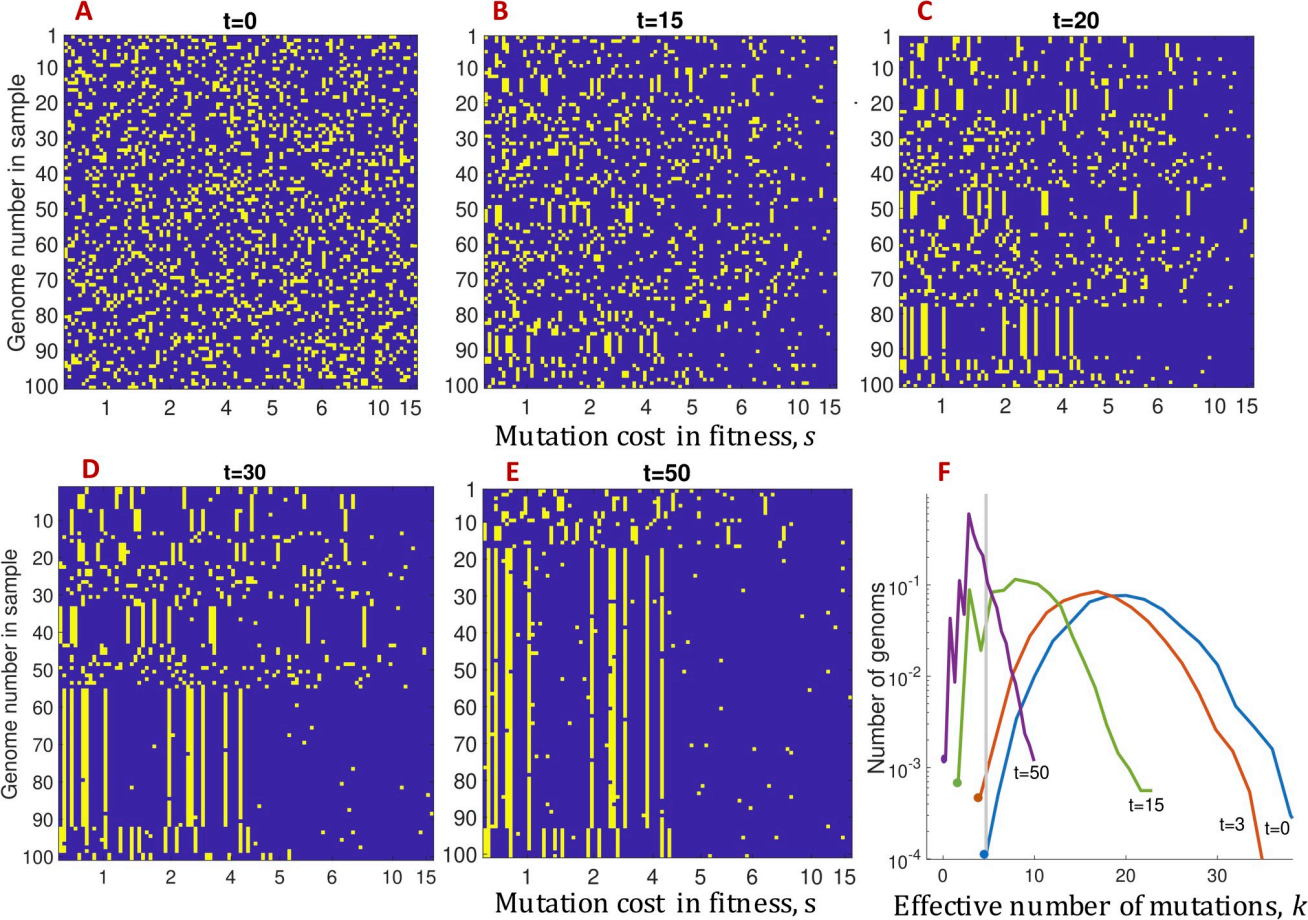
Deleterious alleles with higher values of fitness cost, *s*, are the first to be depleted during the process of adaptation (single run). (A-E) Evolution of a sample of 10^2^ sequences. Violet dots: better-fit alleles, yellow dots: less-fit alleles. X-axis: the cost in fitness, *s*, multiplied by 100. The values of *s* are randomly distributed with the half-Gaussian distribution, *s*>0, with the average *s*_*av*_ = 0.05. Genomic sites are ordered by the value of *s*. Y-axis: genome number in the sample. The initial population is randomized with the average frequency of deleterious alleles *f*_*in*_ = 0.2. Time points in generations are shown. (F) Evolution of the genome distribution in fitness. X-axis: the effective number of deleterious alleles, defined as *k* = −*W*/*s*_*av*_. where *W* is genome fitness. Different colors show discrete time intervals from 0 to 5. The Vertical grey line shows the most-fit class of genomes at *t* = 0. The emergence of clonal structure in (A-E) coincides with the transition from the selection of pre-existing sequences to the traveling wave regime. The other parameters: population size *N* = 10^4^, number of sites *L* = 100, genomic mutation rate *μL* = 0.05. In the text, we study the ensemble-average allelic frequency averaged over many runs.

We start from an initial population of *N* genomes that has a fraction of deleterious alleles randomly distributed among genomic sites and genomes (Fig 2A). Evolution of a sample of hundred sequences in a representative Monte-Carlo run is shown in Fig 2. For the sake of visual convenience, we have re-ordered genomic sites in the ascending order of the value of selection coefficient *s*_*i*_. In our simulations, selection coefficients are chosen randomly at each site from the half-normal distribution

$$g(s)=\frac{2}{s_{av}\pi }\exp\left(-\frac{s^{2}}{\pi {s_{av}}^{2}}\right)$$ (4)

where *s*_*av*_ is the average mutation cost in fitness for the initial state.

In the process of evolution, we observe an increasing redistribution of deleterious alleles among genomic sites, as follows (Fig 2). The sites with a relatively high mutation cost loose deleterious alleles due to natural selection. The asymmetry becomes evident from *t* = 20. Finally, at *t* = 50 (Fig 2E), mutations on the right-hand side are almost absent. Thus, deleterious alleles with higher values of mutation cost vanish first, which explains qualitatively the observed exponential dependence of DFE on *s* (Fig 1). The log-slope increases in time.

We note that, in our example, we used a rather large initial value of deleterious allele frequency, *f*_*in*_ = 0.2, which is convenient for numerical computation. In real life, mutant frequency *f* may be much smaller. However, our main conclusions do not depend on this initial condition. Below, we use analytic derivation that applies to any low *f* far above the equilibrium value.

In Fig 2, two intervals of adaptation can be discerned. Early on, new beneficial mutations can be neglected, and the critical evolutionary factor is the natural selection of pre-existing alleles (Fig 2A and 2B). This fact was demonstrated experimentally in the evolution of vesicular stomatitis virus in culture [37]. In time interval, *t*≪1/*s*_*av*_, where *s*_*av*_ is the average of *g*(*s*), the distribution of alleles over genomes remains random. In contrast, in the second time interval, which starts around *t*~1/*s*_*av*_, we observe deleterious alleles spanning large groups of genomes. In this regime, new beneficial mutations become crucial for further evolution, because they give birth to new highest-fit genomes (Fig 2B–2E). To explain the formation and subsequent growth of groups of identical sequences, clones (Fig 2B–2E), we use the traveling wave theory (Fig 2F).

Formation of these clones occurs at the edge of the traveling wave of fitness distribution [15,16,23,24] (Fig 2F). The fitness distribution moves in time towards higher values of fitness, i.e., smaller numbers of deleterious alleles. At early times, the distribution is broad and symmetric. In this regime, as was mentioned earlier, the main force is the selection of preexisting genomes. After a while (*t*~1/*s*_*av*_), the profile becomes asymmetric, and the high-fitness edge starts to move to the left together with the peak due to new beneficial mutations (Fig 2F). The genomes, appearing on the left side from the initial high-fitness edge (grey line in Fig 2F) share the initial genetic background. Hence, they produce the observed groups of sequences identical at most sites (yellow vertical lines, Fig 2B). As the wave progresses, the clonal structure grows, and eventually, most genomes in the population become an offspring of the same ancestor (Fig 2E).

### Analytic derivation of universal DFE

In this section, we study analytically a general non-equilibrium case of slow adaptation. We also assume that mutant frequency, which we study as the ensemble-average, 〈*f*(*s*,*t*)〉, has evolved for some time before the experiment measuring DFE, but that the population is not in equilibrium yet, so that deleterious mutation events (reverse mutations) are negligible. Below, we present the results of three independent derivations for three limiting cases, as follows. The detailed derivations are in *Materials and Methods*.

- The case where 〈*f*(*s*,*t*)〉 is dominated by polymorphic sites (short-term evolution, Fig 2A and 2B),
- the case where 〈*f*(*s*,*t*)〉 is dominated by monomorphic less-fit sites (moderate-term evolution, Fig 2D and 2E),
- the general case, where both components can be important. In all three derivations, 〈*f*(*s*,*t*)〉 is found to be an exponential in *s*, which shrinks in time (see data in Fig 1).

#### Early evolution

We start from the case of a high initial polymorphism, which occurs when a diverse population migrates into another, very different environment, or the external conditions have changed. As simulation in Fig 2 illustrates, the evolution of genomes on the short time scale, *t* ≪ 1/*s*_*av*_, occurs due to the selection of preexisting variation, and new mutation events are not important. Almost all sites are polymorphic. The probability of having a deleterious allele at a polymorphic site with mutation cost *s* at time *t* has the form (*Materials and Methods*)

$$\langle f(s,t)\rangle =\frac{f_{in}}{(1-f_{in})e^{ts}+f_{in}}$$ (5)

where *f*_in_ is the initial mutant frequency.

The log-slope of the distribution of deleterious alleles is defined as

$$\beta =-\frac{\partial \log\left(\langle f\rangle \right)}{\partial s}$$ (6)

**Fig 3:**
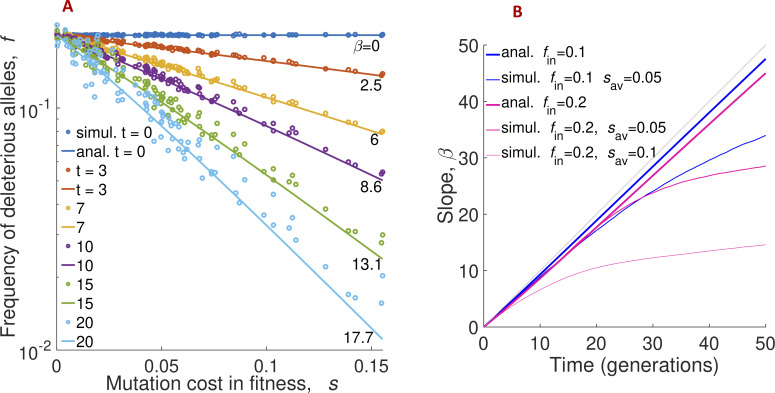
The frequency of deleterious alleles decays exponentially with their fitness effect, with the slope increasing in time. (A) Analytic prediction for the frequency of deleterious alleles from Eq 5 agrees with Monte-Carlo simulation. X-axis: Mutation cost of deleterious allele at a genomic site, *s*. Y-axis: Frequency of deleterious alleles at such a site, *f*(*s*). The mutant frequency *f* is averaged over 20 random simulation runs, the straight lines are linear regression. Different colors show different times, symbols are simulation, and lines are analytic prediction (Eq 5). The numbers on the curves are the values of the slope. Parameters as in Fig 2. (B) The slope of the distribution of deleterious alleles *β*, analytic (blue lines) and simulation (purple lines), as a function of time, *t*. Parameters *f*_*in*_ and *s*_*av*_, if different from those in Fig 2, are shown on the legend. The log-slope for the simulated curves of mutant frequency in (A) is obtained by an exponential fit. We observe that the deviation of the simulated slope from the analytic prediction Eq 5 at long times coincides with the establishment of the traveling regime, which occurs later for smaller *s*_*av*_ (Fig 2F). At long times, the traveling wave prediction, Eq 8, applies (dashed blue lines). Grey diagonal shows *β*(*t*) = *t*. Parameters are as in the legend of Fig 2.

We observe that the formula in Eq 5 does not depend on the initial distribution of selection coefficients among sites, *g*(*s*). At a small initial mutant frequency *f*_*in*_, the formula can be approximated with an exponential, *f*(*s*,*t*) ≈ *f*_*in*_exp(−*ts*). The exponential slope is approximately equal to time, *β* = *t* (Fig 3).

#### Traveling wave regime

At longer times, *t* ≫ 1/*s*_*av*_, beneficial or deleterious alleles become fixed at many sites, and the above derivation does not apply. We need to use the results of the traveling wave theory, which takes into account the effects of selection, linkage (clonal interference and background selection), and random drift. In the stationary regime of the traveling wave (Fig 2F), fixation of new beneficial alleles is the process that dominates the loss of deleterious alleles [15,16,23,24]. In each realization, most sites are monomorphic, either in the less-fit allele or in the better-fit allele. Locations of fixed and variable sites differ strongly between realizations. The average less-fit allele frequency, 〈*f*(*s*,*t*)〉, is dominated by the frequency of less-fit monomorphic sites (not polymorphic sites). Their number decreases in time, as new beneficial alleles are being added to the population and fixed. To obtain an equation for 〈*f*(*s*,*t*)〉, we use the previously calculated fixation rate of beneficial alleles [24], as follows.

Let *t*_0_ ~ 1/*s*_*av*_ be the characteristic time when the traveling wave regime starts. In *Materials and Methods*, we solve a dynamic equation for the ensemble-averaged allelic frequency, 〈*f*(*s*,*t*)〉, which can be expressed in terms of the fixation rate of new beneficial mutations, as follows

$$\langle f(s,t)\rangle =f_{in}e^{-t_{0}s-\mu N\int_{t_{0}}^{t}\varphi (s)dt}$$ (7)

where *φ*(*s*) is the probability of fixation of a beneficial mutation with fitness gain *s* derived previously [24] (see *Materials and Methods*). The linkage effects enter the problem through *φ*(*s*), which is generally smaller than its one-locus value, *φ*(*s*) = *s*. The assumption here is that an established allele that does not become extinct is always fixed in the end. By expanding the argument of the exponential in Eq 7 in *s*, the slope takes the form

$$\beta (t)=t_{0}+\mu N\varphi \prime (0)(t-t_{0})$$ (8)

where *φ*′(0) is derived in *Materials and Methods*. We explain how to estimate *t*_0_ from the simulation below.

#### Quasi-equilibrium argument

We now compare these two cases with a more general argument [12]. As it has been tested by simulation in a realistic parameter range [12], because the wave is slow, the system is in quasi-equilibrium, where all the variables change slowly adjusting to the slow change of the average fitness in time. The slow process is the fixation of rare mutations at the leading edge of fitness distribution at *NU*_*b*_ ≫ 1. While the distribution is crawling forward in fitness, each fitness class has enough time to reach equilibrium driven by random drift. The progress of the wave is slow compared to equilibration of fitness classes. Hence, given the fitness distribution of genomes, the distribution of alleles over sites and genomes is determined by the condition that entropy is maximal. (However, the full equilibrium does not occur until much later on time scales much larger than 1/<*s*>.) Furthermore, in the traveling wave regime, the fitness distribution is narrow, Δ*W* ≪ |*W*|. Therefore, system’s entropy is at the conditional maximum, *S*(*W*), restricted by the average value of fitness, *W*. (This state is not to be confused with Quasi Linkage Equilibrium observed at large recombination rates in the presence of epistasis).

The maximum of entropy is a general property of chaotic systems. It is well-known from statistical physics that entropy reaches maximum in the long run. The same is correct for systems that change slowly. The simplest way of looking at it is that a chaotic system assumes the most probable state [38].

From this consideration, the probability to have a deleterious allele at a given site is given by [12]

$$\langle f(s,t)\rangle ={[1-\langle f(s,t)\rangle ]e}^{-\beta (t)s}$$ (9)

where $\beta =\frac{\partial S}{\partial W}$. Eq 9 is general, while Eqs 5, 6, and 8, provide explicit expressions for *β*(*t*). Again, we remind that *β*(*t*) depends on time, because the population is not in true equilibrium but in the process of slow adaptation.

The mathematical form of Eq 9, called "Fermi statistics", is well-known for equilibrium systems. For the first time, it was obtained by Enrico Fermi from Boltzman distribution for the statistics of a specific type of quantum particles, where each energy level can be either empty or filled with only one particle. In biology, Fermi distribution for Eq 9 has been obtained for various two-state systems in equilibrium [39–41].

We remind that our results apply in the broad intermediate region of times, *t* > 1/*s*, in the adaptation regime, when the traveling wave has already been established, but the equilibrium has not been reached yet. One group [41] have previously considered a similar model, but in the steady-state case where deleterious and beneficial mutations balance. They have derived a similar expression for another quantity, the ratio of densities of beneficial and deleterious mutations (see their Eq 9). In the non-neutral limit, they obtained *β* = *T*_2_, the time to most common ancestor for a pair of sequences. In contrast, we consider the case of adaptation far from the steady state, where deleterious mutation can be neglected. As we can observe, the exponential slope, Eq 8, depends on time, as it should during adaptation process and differs from *β* = *T*_2_ obtained in steady state [41]. Thus, taken together, these two results demonstrate that exponential dependence holds both far from the steady state (our work) and near steady state (Rice, et al. 2015).

The reason for the non-trivial validity of Fermi statistics far from equilibrium, in our case, is the adiabatic (quasi-equilibrium) regime, which exist in a broad parameter region in the traveling wave regime [12,13,35]. This regime applies even for slowly changing selective pressure, such as exists in the case of influenza due to accumulation immune memory cells. This case can be mapped onto the traveling wave for constant selection condition [42,43]. The crossover region between the adaptation regime we study and full equilibrium is not amenable to our analytic method, which neglects mutations. It requires a separate analysis.

Thus, Eqs 5 to 9 demonstrate that the exponential dependence on *s* in 〈*f*(*s*,*t*)〉 arises in the course of evolution at any initial conditions after the evolution time *t* ~ 1/*s*_*av*_, and that the resulting exponential slope is robust to the initial conditions.

### Monte-Carlo simulation confirms theory

To test our analytic theory, we compare the frequency of deleterious alleles, 〈*f*(*s*,*t*)〉 in Eq 5, with the results of Monte-Carlo simulation averaged over 20 random runs at several time points (Fig 3A). At *t* = 0, the simulated and analytic 〈*f*(*s*,*t*)〉 do not depend on *s*, because all sites are assumed to have the initial frequency of deleterious alleles, *f*_*in*_. Thus, the slope *β* is equal to 0 (blue line). At later times, we observe that the slope increases gradually in time, and function 〈*f*(*s*,*t*)〉 depends exponentially on *s*. Apart from some residual fluctuations, our analytical formula (Eq 5) demonstrates good agreement with simulation. Because sites with mutation gain *s* are the sites with deleterious alleles with fitness cost *s*, we confirm that the distribution of beneficial fitness effects maintains an exponential shape in a broad interval of time.

Next, we compare the analytic prediction for the log-slope, *β*, given by Eqs 5 and 6, with simulation results for different values of the average selection coefficient, *s*_*av*_ (Fig 3B). We observe a good match at early times. The results are not very sensitive to the variation of the initial allele frequency, *f*_*in*_, or the other model parameters (Fig 3B). At longer times, *t* > 1/*s*_av_, Eqs 5 and 6 and simulation results diverge. This is caused by entering the traveling wave regime. In this regime, the distribution in fitness moves beyond the best-fit sequence present in the initial population due to beneficial mutations *de novo* (Fig 2F). To predict the slope analytically, we need to account for the effect of new beneficial mutations [24], as it is done in Eqs 7 and 8. To connect the two time intervals, we adjust the value of *t*_0_ in Eq 8 to make simulated and analytical curves match each other. Fitting with a single parameter matches the heights of segments in Fig 3B but does not affect the time derivative *dβ*/*dt* in the traveling wave regime, *t* ≫ *t*_0_, so it can be meaningfully compared to simulation. We observe a good agreement in *dβ*/*dt* between simulation and the analytic prediction, Eq 8 (Fig 3B). To summarize this section, our model of evolution provides an explanation for the exponential form of DFE (Fig 1).

### Calculating selection coefficients from a protein or nucleotide sequence set

Our results have practical application. They enable us to infer the relative values of the selection coefficient, *s*, using sequence sets at several time points, as long as the system is still adapting. For the method to work, the system has to satisfy several requirements, based on the assumptions of the model, as follows:

- Selection type is directional and constant (or, at least, changing slowly on scale *t* ~ 1/*s*).
- Multiple samples from replicate populations are available to calculate 〈*f*(*s*,*t*)〉.
- The organism or the virus is well-evolved in the past but not in the mutation-selection equilibrium yet, 〈*f*(*s*,*t*) 〉≫ *f*_*eq*_.
- Epistasis is not included explicitly, because it is assumed to be incorporated in the renormalized values of *s*. This is a good approximation on sufficiently short time scales. On a long time scale, genomes must be described as having many epistatic pairs, and the effective values of *s* change. The inference of epistasis is addressed elsewhere [35].

The proposed method is not sensitive to the fine details of replication cycle, the generation overlap in time, or the statistics of random genetic drift. We start by obtaining a database of aligned sequences of a pathogen or organism at several time points, *t* (at least, two time points). We determine the consensus allele at each aminoacid position as the most abundant variant. Then, we binarize sequences by replacing each consensus allele with 0, and any minority allele with 1. After binarization, we calculate the frequency of 1 for each site, *f*_*i*_(*t*). Insertions and deletions are eliminated. This technique is appropriate if most diverse sites have one or two minority variants.

Based on the analytic results above, the relative value of selection coefficient *s*_*i*_ at aminoacid position *i* can be estimated from

$$\beta (t)s_{i}=-log\left[\frac{f_{i}(t)}{f_{norm}}\right]$$ (10)

The presence of an additional factor *f*_norm_ in Eq 10 is due to the fact that, in Eqs 6 to 9, *f*_*i*_ represents the average frequency of less-fit alleles at site *i*, hence, *s*_*i*_ > 0 for all sites, by the definition. In real sequences, the best-fit sequence is not known and usually approximated with the consensus sequence. Hence, an anti-consensus allele can be better-fit at some sites, as given by *s*_*i*_ < 0. Since some sites will have negative *s*_*i*_, we need to introduce normalization constant *f*_norm_, and such sites have *f*_*i*_ > *f*_norm_.

Factor *f*_norm_ is estimated, as follows. The left-hand side in Eq 10 factorizes into a product of two terms: one depends only on time, and another only on site number *i*. This fact implies the existence of a fixed point in *s*_*i*_ independent on time, *t*, which can be used to determine the normalization factor *f*_norm_, as follows. For each time, we rank genomic sites in the descending order in *s* and map *i*→*m*_*i*_ where *i* is the label of an actual site in genome, and *m_i_* is its number after the ranking in *s*. We obtain a monotonous ranked curve, *s*_rank_(*m*, *t*). Then, we find the intersection between curves *s*_rank_(*m*, *t*) obtained at different times, *t*. Next, we adjust the value of *f*_norm_ in Eq 10 until we obtain *s*_rank_ = 0 at the intersection point.

The resulting estimate of *β*(*t*)*s*_*i*_ from Eq 10 represents the selection coefficient at site *i* in units of 1/*β*. Further, taking the inverse derivative from each ranked *s* curve, we obtain the distribution density of selection coefficient over non-conserved sites, as given by *g*(*s*) = [*δs*/*δm*]^−1^. Finally, we can re-order the ranked sites back, *i*←*m*_*i*_ and plot the relative values of selection coefficient, *βs*_*i*_, against their actual aminoacid positions, *i*.

We note in conclusion that frequency *f*_*i*_(*t*) in analytic Eqs 5 to 9 is assumed to be ensemble-averaged, including realizations where site *i* is monomorphic and realizations where it is polymorphic. Ensemble averaging can be approximated in data by combining sequences from different independent populations (different geographic locations).

## Discussion

We proposed an evolutionary explanation for the exponential DFE(s) of beneficial mutations in terms of the mutation gain in fitness. Using an asexual population model, we predicted a gradual depletion of deleterious alleles with higher fitness costs accompanied by the emergence of a clonal structure after *t* ≈ 1/*s*_*av*_. First, neglecting new mutation events, we obtained an exponential dependence of allelic frequency on fitness. The logarithmic slope of DFE in *s* is equal to time. At longer times, when beneficial mutations become crucial for the generation of new highly fit genomes, we obtained another expression based on the traveling wave theory. Our results confirm previous work [12] where an exponential dependence for deleterious allele frequency was predicted using a general entropy argument.

Based on the experiments cited in *Introduction*, many models assume an exponential distribution of fitness effects as a starting assumption [11,24,25]. Our findings provide an evolutionary justification for this assumption and update these theories by predicting that the distribution is not constant but shrinks in time. However, when mutation-selection balance is approached, reverse mutations demolish selection as well as the exponential dependence in DFE(s). Instead, in this case an exponential dependence was predicted for ratio DFE(*s*)/DFE(−*s*) [41].

Some groups attempted to explain the universality of the exponential DFE using formal statistical arguments, such as the extreme-value theory [44–46]. There are essential differences between this pioneering work and our findings. In the cited work, the aim was to prove an exponential distribution for the raw distribution of selection coefficient among all possible genomic sites, *g*(*s*), in the limit of large *s*.

In contrast, we demonstrate that the exponential dependence of DFE on selection coefficient is mostly determined, in the long run, by allelic frequency 〈*f*(*s*,*t*)〉 in the broad range of *s*. Also, the cited approach [44–46] predicts a constant slope for DFE(*s*), while our analysis, simulation, and the experimental data (Fig 1) prove that it changes in time. Based on our results, we proposed a method to estimate selection coefficients for each diverse site in mutiple sequence sets from two or more time points.

The main limitation of the present approach is that it considers long-term constant directional selection. In real systems, selection can be balancing due to diploid dominance, or it can alternate sign [32], or be imposed by changing external conditions. Examples from virology include the mounting immune response [47] or the interaction between a virus and its defective interference particles [48,49]. All these cases require separate approaches.

To conclude, we demonstrated that the exponential DFE observed in viruses and bacteria arises naturally in the process of adaptation under directional selection. The present paper explains the universal DFE from the first principles of population genetics and proposes a general method to measure the intrinsic spectrum, *g*(*s*). We will consider specific applications to real genomic data elsewhere.

## Materials and methods

### Early evolution

We focus on a genomic site chosen from *L* sites, which selection coefficient, defined with respect to the best-fit possible sequence, is assumed to be known and equal to −*s*, where *s* > 0. The other selection coefficients in the genome are assumed to vary according to a random distribution with density *g*(*s*). We make no assumption regarding the shape of *g*(*s*) and assume only that the distribution of *s* at different sites is independent. We assume also that the initial population has a random distribution of less-fit alleles among sites with average frequency *f*_*in*_. Biologically, this initial condition corresponds to a population put into a new environment.

First, we neglect new mutation events, which is appropriate at early times, when evolution is dominated by natural selection acting on standing variation; we include mutation events later on. Let *I*_0_(*s*, *t*) be the proportion of all possible sequences having allele 0 at a given site with selection coefficient *s*, at time *t*. The fraction of sequences having allele 1 at the site is denoted by *I*_1_(*s*, *t*). Then, the corresponding mutant frequency can be found as a ratio

$$\langle f(s,t)\rangle =\frac{I_{1}(s,t)}{I_{0}(s,t)+I_{1}(s,t)}$$ (11)

Selection causes the decay of the number of each sequence, {*K*_*i*_}, by a time-dependent factor, $e^{-t\sum_{i=1}^{L-1}s_{i}K_{i}}$. The values of *I*_1_(*s*, *t*) and *I*_0_(*s*, *t*) in Eq 11 must be averaged over all possible values of *s* and *K*_*i*_ for all sites *i*

$$I_{0}(s,t)=(1-f_{in})Z$$ (12)

$$I_{1}(s,t)=f_{in}e^{-ts}Z,$$

$$Z=\int_{0}^{\infty }{ds}_{1}\int_{0}^{\infty }ds_{2}\ldots \int_{0}^{\infty }ds_{L-1}\sum_{K_{1}=0}^{1}\sum_{K_{2}=0}^{1}\ldots \sum_{K_{L-1}=0}^{1}\prod_{i=1}^{L-1}p(K_{i})g(s_{i})e^{-t\sum_{j=1}^{L-1}s_{j}K_{j}}$$ (13)

Here the initial probabilities of having less-fit and better-fit alleles are *p*(1) = *f*_*in*_ and *p*(0) = 1−*f*_*in*_, respectively. From Eqs 12 and 13, we obtain Eq 5 in *Results*.

### Traveling wave regime

In the traveling wave regime, which starts around *t* > 1/*s*_*av*_, beneficial mutations have to be included into consideration, because they create new highest-fitting genomes of the population. Let *t*_0_ be the characteristic time of the beginning of traveling wave regime and *φ*(*s*) be the fixation probability of beneficial mutations with benefit *s* [24]. In this regime, most of deleterious alleles are found at uniformly deleterious sites (Fig 2, yellow columns). Hence, their loss occurs mostly due to fixation of new beneficial alleles at these sites. Then, the dynamic equation for the frequency of deleterious alleles for *t* > *t*_0_, *t*_0_ ~ 1/*s*_*av*_, has a form

$$\frac{\partial \langle f(s,t)\rangle }{\partial t}=-\mu N\varphi (s)\langle f(s,t)\rangle$$ (14)

The initial condition for Eq 14 can be obtained from the estimate of *f* in the early time phase where the selection of pre-existing genomes is the dominant process. From Eq 5 we obtain

$$\langle f(s,t_{0})\rangle \approx f_{in}e^{-t_{0}s}$$ (15)

The solution of Eq 14 with this initial condition, Eq 15, has the form of Eqs 7 and 8. Thus, the problem is reduced to the result of a previous work [24]

$$\varphi (s)=A\left[e^{-\frac{s^{2}}{2v}}(\frac{e^{s\frac{x_{c}}{v}}-1}{s})+\frac{e^{\frac{{x_{c}}^{2}}{2v}}}{vx_{c}}\int_{x_{c}}^{\infty }dx\;xe^{-\frac{{\left(x-s\right)}^{2}}{2v}}\right],$$

$$A=\frac{1}{N}{\left[\frac{x_{c}}{v}+\frac{1}{x_{c}}\right]}^{-1}$$ (16)

Here *φ*(0) ≈ 1/*N*, which corresponds to the selectively-neutral limit, *v* is the average rate of adaptation, *x*_*c*_ is the characteristic value of fitness corresponding to the boundary of clonal interference, and *N* is the population size.

For sufficiently large population sizes, which correspond to the multiple-mutation regime (see below), we have $x_{c}^{2}\gg v$ and hence can neglect the second term in Eq 16, which yields

$$\varphi (s)=\frac{1}{N}\frac{v}{x_{c}}e^{-\frac{s^{2}}{2v}}\left(\frac{e^{s\frac{x_{c}}{v}}-1}{s}\right)$$ (17)

Assuming small *s* ≪ *v*/*x*_*c*_ [but *s* can be comparable or larger than the long-term value of 1/*β*(*t*)], we obtain *φ*(*s*) ≈ 1/*N*+*φ*′(0)*s*, where

$$\varphi \prime (0)=\frac{x_{c}}{2Nv}$$ (18)

Parameters *v* and *x*_*c*_ were derived previously for the general evolution model with directional selection. They can be expressed in terms of the initial distribution of mutational effects *before* the evolution period starts, *ρ*(*s*), population size *N*, and the probability of beneficial mutation per genome per generation, *U*_*b*_ [24].

For example, consider Gaussian *ρ*(*s*) in Eq 4 with a characteristic scale *s* = *s*_*av*_, as in the simulation in Fig 2. At large population sizes *N* where the traveling regime applies, such that $v\gg s_{av}^{2}$, or log(*Ns*_*av*_) ≫ log^2^(*s*_*av*_/*U*_*b*_), we get

$$x_{c}=\sqrt{2Vlog(Ns_{av})}$$ (19)

$$x_{c}/v=\frac{1}{\sqrt{\pi }s_{av}}log\left(\frac{s_{av}}{U_{b}}\right)$$ (20)

[see [24], Eqs 19, 20, and 22 with $\sigma \equiv \sqrt{\pi }s_{av}$]. Substituting Eq 20 into 18, we obtain the desired value of *φ*′(0) in Eq 8 in *Results*

$$\varphi \prime (0)=\frac{1}{2\sqrt{\pi }Ns_{av}}log\left(\frac{s_{av}}{U_{b}}\right)$$ (21)
